# Impact of urinary incontinence on female sexual health in women during midlife

**DOI:** 10.1186/s40695-015-0007-6

**Published:** 2015-09-14

**Authors:** Christine M. Chu, Lily A. Arya, Uduak U. Andy

**Affiliations:** Division of Urogynecology, Department of Obstetrics and Gynecology, University of Pennsylvania School of Medicine, 3400 Spruce St., 1000 Courtyard Building, Philadelphia, PA 19104 USA

**Keywords:** Sexual function, Sexual dysfunction, Urinary incontinence, Urge urinary incontinence, Stress urinary incontinence, Coital incontinence, Treatment outcome, Middle aged

## Abstract

Sexual health is important to the self worth, emotional well being, and overall quality of life of women in midlife. However, urinary incontinence, which is prevalent in this population, has a negative impact on sexual function. The purpose of this article is to review the impact of urinary incontinence on female sexual dysfunction and discuss the impact of urinary incontinence treatment on sexual function. We carried out a literature review on the effect of stress urinary incontinence and urgency urinary incontinence on sexual health and physiological response, including coital incontinence, satisfaction, desire, orgasm, frequency, and partner relationships. We examined the literature regarding changes in sexual function related to non-surgical and surgical interventions for incontinence. Overall, though studies are lacking and of poor quality, treatment of incontinence has been shown to improve sexual function. Both pelvic muscle training and midurethral slings have been shown to improve sexual function in those with stress urinary incontinence. In urgency urinary incontinence, evidence indicates improvement in sexual function after treatment with anti-muscarinic medications. Coital incontinence commonly improves with treatment of the underlying incontinence subtype. Although problems related to sexual health are complex and involve both psychological and physical factors, it is important to consider treatment of urinary incontinence as part of management of sexual dysfunction.

## Introduction

Urinary incontinence (UI) is a common condition, with reported prevalence ranging from 28 to 47 % in women during midlife [1, 2]. The risk of incontinence increases incrementally from the age of 40 to 60, with prevalence nearly doubled by age 55 [3]. Common types of incontinence include stress incontinence (urinary leakage with activity that increase intra-abdominal pressure), urgency urinary incontinence (leakage related to urgency and irritative bladder symptoms associated with overactive bladder), and mixed incontinence (a combination of stress and urgency urinary incontinence). Stress urinary incontinence is the most common type of urinary incontinence, accounting for 52 - 65 % of urinary incontinence in women aged 30 to 60 [4]. Treatment of stress UI is primarily surgical, while urgency urinary incontinence, a common problem that may affect 20 % of middle-aged women [5], is mainly treated with non-surgical options. In those with mixed urinary incontinence, the most bothersome and dominant incontinence type is treated first. However, coital incontinence, the leakage of urine during sexual intercourse, may have the most impact on sexual health and commonly occurs in women with any type of incontinence, with an overall prevalence from 11 to 60 % in middle-aged women with UI [6].

UI, even when not directly associated with intercourse, plays an important role in altering behaviors of human sexual function. This is concerning, as sexual health is very important in the overall quality of life and is tied to a woman’s self worth, emotional well-being, and even cognitive function [7]. In a recent report, 86 % of women with urinary incontinence reported that sexual health was an important issue; however, few women with UI will discuss problems with sexual health unless directly asked [8]. Our aim is to review the impact of urinary incontinence on female sexual dysfunction and discuss the impact of urinary incontinence treatment on sexual health of women in midlife.

Given the rarity of major, high-level evidence with regards to UI, treatment, and its relationship to sexual health, we searched for any trials related to these topics, prioritizing randomized controlled trials and prospective studies. We conducted a wide and comprehensive literature search in Pubmed (up to December 2014) on any articles examining overall changes in sexual health (overall subject-described impact). Additionally, we looked for the impact of urinary incontinence and treatment on aspects of the sexual physiologic responses (frequency, libido, desire, arousal, lubrication, orgasm, satisfaction, pain). No date or language restriction was used. Questionnaires and questionnaire subdomains used by studies to evaluate changes in sexual health are briefly described in Table 1.

**Table 1:** Sexual function questionnaires and subscale domains

Questionnaire or Subscale Name	Items	Domains/Descriptions
Female Sexual Function Index (FSFI)	19	Desire, Arousal, Satisfaction, Lubrication, Orgasm, Pain
Bristol Female Lower Urinary Tract Symptoms (BFLUTS): Sex Life Items	4	Urinary related sex life problems (2), Pain, Coital Incontinence
King’s Health Questionnaire (KHQ): Personal Relationship Domain	3	Relationship with partner, Effect on sex life, Effect of family life
Short form Personal Experiences Questionnaire (SPEQ): 3 Domains	3	Libido, Arousal, Dyspareunia
Pelvic Organ Prolapse-Urinary Incontinence Sexual Function Questionnaire (PISQ)	31 Short form: 12	31: Behavioral/Emotive (15), Physical (10), Partner-related (6) Short form: Behavioral/Emotive (4), Physical (5), Partner-related (3)
Beck Depression Inventory II: Question 21	1	Loss of interest in sex
Sexual Quality of Life-Female (SQOL-F)	19	Desire, Lubrication, Arousal, Pain, Orgasm, Satisfaction
Arizona Sexual Experience Scale	5	Desire, Arousal, Lubrication, Orgasm, Satisfaction
Nine questions regarding Sexual Functioning (NSF-9)	9	Libido, Frequency, Lubrication, Orgasm, Time to Orgasm, Pain, Satisfaction
Lemack	8	Presence of pre-operative sexual activity, Frequency, Satisfaction with intercourse, Satisfaction with surgery, Coital incontinence, Partner pain
Electronic Pelvic Floor Symptoms Assessment Questionnaire (ePAQ): Urinary domain	4	Impact of urinary symptoms on sex life, Anxiety, Avoidance of sex, Partner avoidance of sex

## Review

### Urinary incontinence and overall impact on sexual health

Because of the proximity of the bladder and urethra to the vagina and vulva, UI may have major effects on the sexual health of affected women. In clinic settings, incontinent middle-aged women commonly report disruption of sexual health with a median percentage of 28 % [9]. Complete abstinence from sex secondary to urinary incontinence can range from 5.9 to 38 %, a wide range owing to the diversity of the populations included in these studies [10–12]. Older women with urinary incontinence report decreased self-rated health and a greater incidence of depression [5], which may also affect sexual health. Women with UI show greater dysfunction on validated sexual function questionnaires compared to those without incontinence [13], regardless of menopausal status [14].

When examining sexual health in women with stress UI, older age, postmenopausal state, greater prolapse, and greater parity have been associated with worse sexual function scores [15]. The severity and duration of stress UI may not be associated with the level of sexual function [16, 17]. In patients with urgency UI, twenty-five percent of women with urgency UI report negative impacts on their sex life [18, 19]. Urgency UI is also significantly associated with lower self-esteem [20], which significantly influences sexual dysfunction [21].

#### Impact on the female sexual response

Severe UI has been found to be significantly associated with decreased libido and vaginal dryness [22], decreased interest, and decreased satisfaction with sexual intercourse, including orgasmic dysfunction [9].

In particular, women with stress UI have been noted to have problems with desire, arousal, and lubrication [23]. Decreased desire in these women may be related to unsatisfying partner relationship, worries about coital incontinence, and unsatisfying somatic health [21]. In spite of decreased sexual desire, a majority of women with stress UI (78 %) are able to achieve orgasm [16], and may not necessarily display decreased sexual activity [24]. Women with urgency UI similarly also report significant difficulty with hypoactive sexual desire, and arousal disorder; however, achieving orgasm is also difficult in this population [25–27]. In women with urgency UI, this results in decreased sexual activity [28] and decreased sexual satisfaction associated with urgency UI [2].

Dyspareunia, pain with intercourse, often accompanies UI and varies in prevalence from 8 to 42 % amongst middle-aged women with UI [9]. Studies have reported significantly greater rates of dyspareunia in women with UI and other lower urinary tract symptoms compared to controls [22, 25]. Dyspareunia has been noted more frequently in women with stress UI compared to those with overactive bladder [15]. However, dyspareunia does commonly accompany urgency UI [28, 29].

#### Impact on partners’ sexual health

UI also affects the sexual function of partners. Male partners of women with any type of UI report decreased overall sexual function, satisfaction, frequency of intercourse, and increased rates of erectile dysfunction as compared with partners of women without UI [30]. In spite of these negative effects, 42 % were unaware of the presence of coital incontinence. Of those who were aware, the majority (65 %) did not consider the UI to be the main problem affecting sexual health [21]. Negative effects on marital relationships have been correlated specifically with the presence of urgency UI [31].

### Coital incontinence and impact on sexual health

Coital incontinence deserves particular focus as it is often directly associated with sexual dysfunction. Coital incontinence is prevalent in up to 56 % of middle-aged women with incontinence [9], and peaks around the age of 50, with subsequent decrease as women enter their sixth decade of life [32]. Risk factors for coital incontinence include severity of incontinence [33], obesity [34], parity [35], and anterior and posterior vaginal wall prolapse [35]. The severity of the coital incontinence may be associated with the degree of sexual dysfunction [35, 36].

The impact of coital incontinence on sexual function is multifold. Actual leakage during coitus can affect sexual satisfaction. However, worry about leakage also contributes, and is significantly associated with decreased sexual desire and sexual satisfaction [21]. Embarrassment, guilt and anxiety about sexual activities are highly prevalent in this population [37]. Arousal can be also compromised, as patients with coital incontinence have significantly greater issues with lubrication [37]. Dyspareunia may be increased in women with coital incontinence compared to those without urinary complaints [37]. Avoidance of intercourse specifically because of coital incontinence is common [37]. Among partners of women with coital incontinence, an association with increased ejaculation before reaching full erection was noted [37].

Coital incontinence may occur at any time during intercourse, but is most commonly noted during penetration and orgasm [21, 38], although it is also frequently reported during clitoral stimulation and arousal [21]. Coital incontinence during penetration is commonly associated with stress UI [35, 39–41], while detrusor spasms have been implicated in coital incontinence during orgasm [39, 42]. Coital incontinence resulting from stress UI during penetration may be due to the alteration of the urethrovesical angle and elevation of the bladder neck by the erect penis during moments of increased intra-abdominal pressure. The mechanism of urinary incontinence during orgasm is unclear. It is postulated that penile stimulation of the nerve rich area of the bladder base and trigone may trigger detrusor overactivity in those with severe overactive bladder [42]. Alternately, stimulation of the vanilloid receptors in this area, which are reportedly increased in density in patients with urgency, may trigger detrusor contractions [43]. However, other studies have found no relationship between timing of coital incontinence and stress UI, detrusor overactivity, or mixed urinary incontinence [38, 44]; and that regardless of timing of coital incontinence, stress UI was the most frequently diagnosed type of incontinence, while detrusor overactivity was uncommon [45].

### The impact of treatment of urinary incontinence on sexual health

Treatment of incontinence includes both non-surgical and surgical modalities. Non-surgical treatment for stress UI includes pelvic muscle training (strengthening the levator ani muscles, the main support for the bladder during increased intra-abdominal pressure) [46], anti-incontinence pessaries, and transvaginal electrical stimulation. However, the treatment of stress UI is primarily surgical, and includes midurethral mesh slings, Burch colposuspension, and periurethral bulking injection (injection of bulking agents into the urethral wall to improve continence) [47]. Urgency UI is typically treated by non-surgical methods, including pelvic muscle training and biofeedback, transvaginal electrical stimulation, medication, percutaneous tibial nerve stimulation (involving stimulation of sacral plexus via the tibial nerve). Surgical therapies for urgency UI include botulinum toxin injection of the detrusor muscle and sacral neuromodulation (implantation of a device that stimulates the sacral nerve). Treatment of coital incontinence, like treatment for mixed urinary incontinence, is generally based on the treatment of the dominant incontinence type.

In the discussion below, we will address the effect of treatments for stress UI and urge UI on sexual function, the physiological female sexual response, and coital incontinence.

### Non-surgical treatment of urinary incontinence

Table 2 summarizes the effect of non-surgical treatments for stress UI and urgency UI on sexual function.

**Table 2:** Effect of non-surgical treatments for urinary incontinence on sexual function

Study	Study Design	N	Treatment	Treatment Length	Instrument	Findings	
						Sexual Function	Coital Incontinence
Pelvic Floor Muscle Training (SUI)							
Bo et al [48]	Randomized controlled trial	1. PMFT: 25	PMFT	6 months	B-FLUTS	• Non-significant improvement in pain, urinary-related sexual problems	• Improved
		2. Control: 30					
Zahariou et al [49]	Prospective case series	Total: 58	PFMT	12 months	FSFI	• Improvement in FSFI and subscale scores (*p* < 0.05)	• Improved (*p* < 0.005)
Pelvic Floor Muscle Training (UUI)							
Wang et al [50]	RCT	1. PFMT: 34	1. PFMT	12 weeks	KHQ: Personal Relationship Domain	• Non-significant improvement in Personal Relationship Domain in biofeedback-assisted PFMT group	N/A
		2. Biofeedback-assisted PFMT: 34	2. Biofeedback-assisted PFMT				
		3. Transvaginal electrical stimulation: 35	3. Transvaginal electrical stimulation				
Anti-incontinence Pessary (SUI)							
Handa et al [52]	Randomized controlled trial	1. Continence pessary: 149	1. Continence pessary	12 months	SPEQ (3 domains), PISQ-12	Responders (vs. non-responders):	• Improved (*p* = 0.0002)
		2. Behavioral therapy: 146	2. PMFT			• PISQ improved (*p* = 0.007)	• Greater improvement
		3. Combo: 150	3. Combination			• Restriction of sex due to UI improved (*p* = 0.008)	• with combo therapy (*p* = 0.019) and behavioral (*p* = 0.02) vs. pessary alone
						• Dyspareunia improved (*p* = 0.017)	
Transvaginal electrical stimulation (SUI and UUI)							
Giuseppe et al [51]	Prospective case series	Total: 23	Transvaginal electrical stimulation	3 months	FSFI	Significant improvement in FSFI and all subscale scores (p ≤ 0.01) except arousal, orgasm	N/A
		SUI: 8					
		UUI: 10					
		MUI: 5					
Anti-cholinergic medication (UUI)							
Sand et al [53]	RCT	Total: 2878	1. Patient education & transdermal oxybutynin	12 weeks	KHQ: Personal Relationship Domain	Significant improvement in KHQ score, bladder pain, effect of OAB on sex life, interest in sex	Improved
		Female: 2508	2. Transdermal oxybutynin only				
Rogers et al [54]	RCT	1. Placebo: 189	1. Placebo	12 weeks	PISQ	Significant improvement of PISQ and domain scores after 12 weeks	N/A
		2. Tolterodine: 188	2. Tolterodine 4 mg ER daily			Stable but no continued improvement if used 12 additional weeks except in Physical domain	
Danilova et al [58]	Prospective case series	57	Trospium 15 mg three times daily	16 weeks	Unknown	Sexual dysfunction decreased	N/A
Chapple et al [56]	RCT	1. Placebo: 283	1. Placebo	12 weeks	KHQ: Personal Relationship Domain	Patients with OAB, total	N/A
		2. Fesoterodine 4/8 mg: 272	2. Fesoterodine 4 mg			Statistically significant improvement in fesoterodine 8 mg (vs. placebo) (mean score change of -11.9 v -6.2, *p* < 0.05)	
		3. Tolterodine ER 4 mg: 290	3. Fesoterodine 8 mg			Patients with both UUI & OAB:	
			4. Tolterodine ER 4 mg			Statistically significant improvement intolterodine (vs. placebo) (-12.7 v -6.8, p < 0.05)	
Percutaneous tibial nerve stimulation (UUI)							
Eftekhar et al [59]	RCT	1. Percutaneous tibial nerve stimulation and tolterodine 4 mg daily: 25	1. Percutaneous tibial nerve stimulation and tolterodine 4 mg daily	12 weeks	FSFI	Within each arm:	N/A
		2. Tolterodine 4 mg daily: 25	2. Tolterodine 4 mg daily			Significant improvement in FSFI and subscale scores after 12 weeks (*p* < 0.001)	
						Between arms:	
						No significant difference in FSFI, subscale scores	
van Balken et al [60]	Prospective case series	Total: 121	Percutaneous tibial nerve stimulation	12 weeks	NSF-9	Significant improvement in Satisfaction (*p* < 0.005), Frequency (*p* < 0.005), Orgasm (*p* < 0.05)	N/A
		Female: 76				No significant change in dyspareunia, lubrication	

*B-FLUTS* Bristol female lower urinary tract symptoms; *FSFI* Female sexual function index; *PISQ-12* Short form pelvic organ prolapse-urinary incontinence sexual function questionnaire; *PFMT* Pelvic floor muscle training; *RCT* Randomized controlled trial; *SA* Sexual activity; *SPEQ*: Short form personal experience questionnaire

In stress UI, several high level, large trials support the idea that pelvic floor muscle training can significantly decrease urinary-related sexual problems [48] as well as improve sexual physiological response in the areas of desire, arousal, lubrication, orgasm, and satisfaction [49]. These improvements may be correlated to increased pelvic muscle strength [49]. Coital incontinence was found to be improved with muscle training [48, 49]. Pelvic muscle training, in combination with biofeedback and occasionally transvaginal electrical stimulation, is also used to treat urgency UI. The effect on sexual function in this population has not been extensively studied, and involves mostly small case series. A small randomized controlled trial comparing traditional pelvic exercises to biofeedback-assisted exercises and transvaginal electrical stimulation found that biofeedback-assisted pelvic floor muscle training resulted in greatest improvement in the King’s Health Questionnaire Personal Relationship domain [50].

Transvaginal electrical stimulation can be used to treat stress and urgency UI through strengthening of the pelvic floor muscles. One case series that included 12 women with stress UI and sexual dysfunction prior to treatment reported statistically significant improvements in overall sexual health (as indicated by improvement in Female Sexual Function Index score) and most subscale domains related to physiological response after 3 months of transvaginal electrical stimulation therapy [51]. Likewise, this modality can be used to treat urgency UI. In the same case series, which included ten women with urgency UI, transvaginal electrical stimulation also led to improved scores in the subjects with urgency UI [51]. A small randomized controlled trial found that transvaginal electrical stimulation resulted to less improvement in the King’s Health Questionnaire Personal Relationship domain than pelvic muscle training [50].

Anti-incontinence pessaries are commonly used for stress UI. One randomized controlled trial comparing anti-incontinence pessaries, pelvic floor muscle training/continence strategies, or both found no difference in the rates of incontinence, overall sexual function improvement, or sexual response achieved by pessaries compared to pelvic muscle training. Pessary users who had improvement in urinary symptoms (58.8 %) had greater improvement in overall sexual health (as indicated by higher Pelvic Organ Prolapse/Incontinence Sexual Questionnaire scores) than those who did not (2.26 ± 3.24 versus 0.48 ± 3.76, *p* = 0.0007) [50]. However, pelvic muscle training may be potentially more effective alone or in combination with anti-incontinence pessaries than with pessaries alone [50].

Anticholinergic medications are one of the first-line treatments for urgency UI. Though these medications are well studied, many large trials do not include specific assessment of changes in sexual function. Oxybutynin and tolterodine are two anti-cholinergic medications with high-quality evidence supporting improvement in sexual function with use. In the Multicentre Assessment of Transdermal Therapy in Overactive Bladder with Oxybutynin (MATRIX), which included 2878 subjects, 19.1 % of women reported improvement in sex life while 11.2 % reported worsening following 6 months of treatment with oxybutnin [52]. Similar proportions of women reported improvement in partner relationships and sexual desire [52]. Treatment with tolterodine was found to improve overall sexual health (higher sexual function questionnaire scores) compared with baseline in two studies, with particular improvement in desire, arousal, orgasm, lubrication, and satisfaction subscales [53, 54]. Fesoterodine likewise was found to improve subjects’ Personal Relationship scores as compared to controls [55]. Other smaller prospective studies have reported some improvement in sexual health following treatment with solifenacin [56] and trospium [57]. Certain trials also found that anti-muscarinic medication may improve coital incontinence. In the MATRIX study, oxybutynin was found to decrease the incidence of coital incontinence from 22.8 to 19.3 %, a statistically significant change [52]. In a study with tolterodine, 59 % of patients with incontinence at orgasm had improvement in response to tolterodine, though they were less likely to respond to treatment compared to those without coital incontinence (41.2 % v 17 %, *p* = 0.023) [39].

There are currently no studies evaluating the effect of a newer class of medication, beta-3 agonists, on sexual health.

Two small, poor quality trials on percutaneous tibial nerve stimulation found mixed sexual health outcomes. In two studies that included middle-aged women, one found no improvement in Female Sexual Function Index scores [58], while the other noted improvement in the Nine Questions Regarding Sexual Functioning scores, particularly in satisfaction, frequency, and orgasm [59].

Overall, there is strong evidence that pelvic muscle training can significantly improve sexual health in women with stress and urgency UI respectively. Transvaginal electrical stimulation (for both stress and urgency UI), pelvic muscle training (urgency UI), and anti-cholinergic medication (urgency UI) may improve sexual health, but data is limited by quantity and quality. Literature on anti-incontinence pessaries (stress UI) and percutaneous tibial nerve stimulation (urgency UI) show mixed improvement in sexual health; more trials are needed.

### Surgical treatment of urinary incontinence

Surgical treatment of stress and urgency UI differ, but both seem to result in overall improvement in sexual health. Table 3 summarizes the effect of surgical treatments of stress UI on sexual function as reported in prospective studies. Midurethral slings are the gold standard for treatment of stress UI in middle-aged women, making up the majority of incontinence procedures performed on women aged 18 to 64 [47]. The effect of midurethral slings on sexual health is supported by several large prospective trials and randomized controlled trials.

**Table 3:** Effect of surgical treatments of stress urinary incontinence on sexual function

Study	Design	N	Treatment	Length of Follow Up (months)	Instrument	Findings				Coital Incontinence
						Overall Post-Operative Sexual Function	Improved	Worsened	No Difference	
Midurethral sling: Retropubic (TVT)										
Jha et al [62]	Prospective case series	62	TVT	3	ePAQ	N/A	• Impact of LUTS on sex (*p* < 0.001)	N/A	• Partner avoidance of sex (*p* = 0.06)	• Improved (*p* < 0.001)
							• Avoidance of sex (*p* < 0.001)			
							• Anxiety of UI & sex (*p* < 0.001)			
Ghezzi et al [70]	Prospective case series	53	TVT	6-12	PISQ	• Improved: 34 %	N/A	N/A	• Fear of incontinence	• Improved in 87 %, with associated improvement:
						• Worsened: 3.8 %			• Frequency	• Frequency
										• Fear of CI
										• Embarrassment
Midurethral sling: Retropubic (TVT) versus Transobterator (TVT-O, TOT) comparative studies										
Elzevier et al [71]	Prospective cohort	1. TVT-O: 34	1. TVT-O	3-4	Lemack	• Improved: 20.6 & 18.2 % (TVT, TVT-O respectively)	N/A	• Dyspareunia from vaginal narrowing in TOT [vs TVT-O] (*p* = 0.026)	• Frequency	• Improved
		2. TOT: 44	2. TOT			• Worsened: 5.9 & 18.2 % (TVT, TVT-O respectively			• Lubrication loss (*p* = 0.612)	
									• Clitoral lumescence reduction, sensibility (*p* = 0.191, *p* = 0.346 respectively)	
Jha et al [64]	Prospective cohort	1. TVT: 43	1. TVT	6	PISQ	N/A	• Total PISQ (*p* < 0.001)	N/A	• Behavior emotive subdomain (*p* = 0.7)	• Improved (*p* < 0.002)
		2. TVT-O: 11	2. TVT-O				• Partner, Physical subdomains (*p* = 0.002, *p* < 0.001 respectively)			
Zyczynski et al [67]	RCT	1. TVT: 298	1. TVT	24	PISQ	N/A	• Total PISQ (*p* < 0.0001)	• PISQ in surgical failure [vs success] (*p* = 0.009)	• PISQ between TVT & TOT	• Improved (*p* < 0.0001)
		2. TOT: 299	2. TOT				• Pain (*p* = 0.003)		• Proportion of sexually active patients post-op	
							• Fear of UI & sex (*p* < 0.0001)			
Filocamo et al [61]	Prospective cohort	1. TVT: 28	1. TVT	12	FSFI	N/A	• Total FSFI (*p* < 0.002)	N/A	• FSFI between TVT & TOT	N/A
		2. TOT: 105	2. TOT				• All subdomains (*p* < 0.002)			
							• Sexual dysfunction (*P* = 0.05)			
Cayan et al [75]	Prospective cohort	1. TVT or vaginal repair: 53	1. TVT or vaginal repair	1. Sling: 32.1 +/- 13.7	FSFI	• Improved: 24.5 & 12.2 % (TVT, Burch respectively)	N/A	• Total FSFI (*p* < 0.001)	• Pain (*p* = 0.162)	N/A
		2. Burch: 41	2. Burch	2. Burch: 35.7 +/- 16.7		• Worsened: 47.2 & 63.4 % (TVT, Burch respectively)		• Desire, arousal, lubrication, orgasm, satisfaction subdomains (p ≤ 0.002)		
								• Total FSFI, desire, arousal, lubrication, orgasm in Burch [vs TVT] (*p* = 0.004-0.026)		
Periurethral bulking injection										
Leone Roberti Maggiore et al [76]	Prospective case series	29	Polyacrylamide hydrogel periurethral injection	12	PISQ, Global sexual satisfaction VAS score	N/A	• Total PISQ (*p* < 0.001)	• Global sex satisfaction in surgical failure [vs success] (*p* < 0.001)	• Pain (*p* = 0.244)	• Improved (*p* < 0.001)
							• Desire, orgasm frequency, excitation, satisfaction, fear of UI & sex, negative emotional reaction, orgasm intensity (*p* < 0.001)			
							• Global sex satisfaction VAS score (*p* < 0.001)			

*CI* Coital incontinence; *ePAQ* Electronic Pelvic Floor Symptoms Assessment Questionnaire; *FSFI* Female sexual function index; *PISQ* Pelvic Organ Prolapse-Urinary Incontinence Sexual Function Questionnaire; *RCT* Randomized controlled trial; *SA* Sexually active; *TVT* Tension-free vaginal tape; *TVT-O* Tension-free vaginal tape - obturator; *TOT* transobturator tape; *VAS* visual analog scale

There is a trend towards improvement in sexual health after correction with a midurethral sling. A meta-analysis of 21 studies noted that the pooled chance of improvement of sexual health following sling placement was 33.9 %, with improvement ranging from 1.8 to 94 % (0.95, 95 % CI 0.34, 1.56) [44]. One study that included 133 middle-aged women reported that 40 % of non-sexually active women reestablished intercourse after surgery [60].

Several studies reported improvement in aspects of female sexual response. There was an association between midurethral sling and decreased anxiety [61], resulting in improvement in sexual spontaneity, arousal, and orgasm in certain patients [62]. However, when examining other specific subdomains, most studies found no changes in sexual desire [63, 64], orgasmic capabilities [63, 64], intercourse frequency [63, 65], or satisfaction [63, 64] following sling surgery. Zycynski et al reported improvement in dyspareunia rates in a group of 406 subjects aged 52.9 ^+^/_-_ 11.0 years following sling surgery for stress UI [66].

Anti-incontinence surgeries can be very effective in treating coital incontinence. In fact, improvement after surgery was primarily attributed to decreased urinary-related sexual complaints such as coital incontinence [32, 61]. Pooled data on midurethral slings from a meta-analysis showed a significant reduction in coital incontinence with an OR 0.12 (CI 0.08-0.17) [44]. Post-operatively, patients also reported decreased fear and embarrassment of coital incontinence [67]. Women with coital incontinence and stress UI at baseline were also more likely to display improvement in frequency and enjoyment of intercourse as compared to those without coital incontinence (32.5 % v 6.8 %) [68].

Worsening of sexual function after midurethral sling placement is less common but possible, approximately 13.1 % in a meta-analysis by Jha et al [44]. This can manifest as new-onset dyspareunia [67], loss of libido [67], and de novo anorgasmia [64, 69]. The mechanism is not understood, but may be attributed to mesh complications such as erosion [70], changes in clitoral blood flow after dissection in the periurethral area, narrowing of the vaginal opening, or potential injury to the pudendal nerve branches [70, 71]. Removal or revision of mesh may improve sexual dysfunction; one case series showed that use of vaginal estrogen or correction of erosion after mesh slings were found to have improvement in all Female Sexual Function Index scores except orgasm [72].

Burch colposuspension is less commonly used to treat stress UI given the availability of minimally invasive techniques. Evidence on the effect of Burch colposuspension on overall sexual health is poor in quality and contradictory. One retrospective study comparing tension-free vaginal tape to Burch found no significant difference in sexual improvement post-operatively, though there was a non-significant increase in worsening of intercourse after tension-free vaginal tape [73]. Another small prospective study found decreased sexual function in both groups, but to a greater degree in patients who underwent Burch colposuspension [74].

Periurethal bulking injections are useful for management of stress UI in women who are poor candidates for general anesthesia, as these procedures can be performed with local anesthetics. Data on the effect of bulking agents on sexual function is limited in number and quality. One small prospective study on 29 patients (mean age: 53 years old) treated with polyacrylamide hydrogel injections reported significant improvement in total Pelvic Organ Prolapse/Incontinence Sexual Questionnaire-12 scores, and 6 patients reestablished sexual activity post-operatively [75]. Sexual response (desire, excitement, and orgasm) likewise improved. Four patients who presented with coital incontinence prior to injections achieved resolution of their incontinence [75].

Table 4 shows the effect of surgical treatments of urgency UI on sexual function. Studies on onabotulinumtoxin A injection and sexual function have not been conducted, though an randomized controlled trial by Nitti et al did note a significant improvement in the King’s Health Questionnaire Personal Relationships domain score following treatment with Botox in women with urgency UI [76]. Data on sacral neuromodulation is mixed. Four low quality studies showed significant improvement in sexual function questionnaire scores [77–80], while one did not [81]. Subscale improvements were noted in lubrication, pain, arousal, satisfaction, and orgasm intensity [77–80]. One case series on sacral neuromodulation indicated decreased coital incontinence in 3 patients, and cured coital incontinence in 2 patients, as well as decreased fear of coital incontinence [79].

**Table 4:** Effect of surgical treatments of urge urinary incontinence on sexual function

Author	Study Design	N	Treatment	Instrument	Findings
Obotulinumtoxin A injection					
Nitti et al [77]	RCT	1. Placebo: 243	1. Placebo	KHQ (Personal Relationship Domain)	• Clinically significant improvement in all KHQ scores, including Personal Relationship (-13.4 v -1.1, *p* < 0.001)
		2. Botox: 249	2. Botox 100 U follow up: 12 weeks		
Sacral neuromodulation					
Zahibi et al [78]	Prospective case series	36	SNM follow up: 6 months	FSFI	• Significant ↑ FSFI total and all subscale scores except desire
					• Pts with voiding dysfunction only: 157 % improvement in total FSFI
Signorello et al [79]	Prospective case series	30	SNM follow up: median 36.3 months	FSFI	• Significant improvement in total FSFI and most domains except orgasm
					• 25 % showed >50 % improvement on total FSFI
Gill et al [80]	Prospective case series	8	SNM follow up: median 3.2 months	FSFI, Female Sexual Health Questionnaire	• Significant improvement in arousal, satisfaction, orgasm
					• 50 % ↓ CI, restriction of SA due to UI
Pauls et al [81]	Prospective case series	7	SNM	FSFI	• Significant ↑ frequency, improvement of FSFI total and desire, lubrication, satisfaction, pain scores
Ingber et al [82]	Prospective case series	27	SNM follow up: 6 months	FSFI	• Non-significant improvement in FSFI in OAB patients (18.6 → 22.4, *p* = 0.257)

*SA* Sexual activity, *FSFI* Female Sexual Function Index; *KHQ* King’s Health Questionnaire; *CI* Coital incontinence; *RCT* Randomized controlled trial; *SNM* Sacral neuromodulation

Overall, there is high quality data to support improvement of sexual function after treatment with midurethral slings. Periurethral injection may improve sexual health, though larger studies are needed. The effect of Burch colposuspension on sexual function is mixed, and more evidence is required. High quality, large studies examining the effect of surgical treatments of urgency UI on sexual function are needed, though sacral neuromodulation shows promise in a few low quality studies.

## Conclusions

Urinary incontinence is a bothersome condition that is prevalent in middle age women. There is significant data to support that urinary incontinence is detrimental to sexual function, especially in women in midlife. While data on the effect of urinary incontinence treatments on sexual function is limited by the lack of large trials and high quality trials, treatment of any incontinence has been shown to improve sexual function. For stress UI, non-surgical and surgical treatments - pelvic muscle training and midurethral slings - have been shown to improve sexual function. For urgency UI, treatment with pelvic muscle training and anti-muscarinic medications has the most evidence of improvement in sexual function. Coital incontinence generally improves with treatment of the underlying incontinence subtype. Though problems with sexual health in middle-aged women with incontinence are admittedly complex, and involve both psychological and physical factors, evaluation and treatment of urinary incontinence is important in the management of this important issue.

## Abbreviations

ATLAS: Ambulatory treatments for leakage associated with stress
UI: Urinary incontinence
